# Villages in the Aging Services Ecosystem: Cases from Metro Boston

**DOI:** 10.1093/geroni/igaf122.2842

**Published:** 2025-12-31

**Authors:** Jennifer Molinsky

**Affiliations:** Harvard University, Cambridge, Massachusetts, United States

## Abstract

In the nearly 25 years since the founding of Beacon Hill Village, over 250 grassroots nonprofit village organizations have been established across the US, providing support and social connection for older adults living in the community, much through mutual support of members and volunteers. There are now multiple villages in the Boston metro area alone, covering geographies ranging from small urban neighborhoods (Beacon Hill Village covers an area approximately one-sixth of a square mile) to multiple municipalities (Cambridge Neighbors serves five communities), from urban to suburban locations. The concentration of villages in a single metro offers a unique opportunity to consider how geography, demographics, and service networks in each community shape villages’ priorities and operations. This session will report on research conducted by the Housing an Aging Society Program at the Harvard Joint Center for Housing Studies Housing focused on village organizations in Beacon Hill, Jamaica Plain, Cambridge, Newton, Wellesley, and Brookline. The research utilizes focus groups and interviews with village staff, members, and local government aging services to gain insight into villages’ place in the aging services ecosystem in the Boston area and how each is responding to the challenges and opportunities of aging in Boston’s high-cost but resource-rich environment. Since villages have relatively small memberships in comparison to the older population in each community and in the region as a whole, we will explore their reach and potential for growth, particularly their capacity to serve less-resourced older adults.

